# Long noncoding RNA AC092171.4 promotes hepatocellular carcinoma progression by sponging microRNA-1271 and upregulating GRB2

**DOI:** 10.18632/aging.103419

**Published:** 2020-07-21

**Authors:** Chengjun Sun, Shanzhou Huang, Yuchen Hou, Zhongqiu Li, Dongmei Xia, Lishan Zhang, Yixi Zhang, Yifeng Cai, Ziming Wang, Qi Zhou, Xiaoshun He, Linwei Wu

**Affiliations:** 1 Organ Transplant Center, The First Affiliated Hospital, Sun Yat-Sen University, Guangzhou 510080, China; 2 Guangdong Provincial Key Laboratory of Organ Donation and Transplant Immunology, Guangzhou 510080, China; 3 Guangdong Provincial International Cooperation Base of Science and Technology, Guangzhou 510080, China; 4 Department of General Surgery, Guangdong General Hospital, Guangdong Academy of Medical Sciences, School of Medicine, South China University of Technology, Guangzhou 510080, China; 5 Department of Liver Surgery, Ren Ji Hospital, School of Medicine, Shanghai Jiao Tong University, Shanghai 200127, China; 6 Department of Biliary and Pancreatic Surgery, The First Affiliated Hospital of Sun Yat-Sen University, Guangzhou 510080, China; 7 Department of Liver Surgery, The First Affiliated Hospital, Sun Yat-Sen University, Guangzhou 510080, China; 8 Department of General Surgery, Hui Ya Hospital, The First Affiliated Hospital, Sun Yat-Sen University, Huizhou 516081, Guangdong, China

**Keywords:** cancer, hepatocellular carcinoma, AC092171.4, epithelial-to-mesenchymal transition, survival

## Abstract

In this study, we investigated the mechanistic role of the long non-coding RNA (lncRNA) AC092171.4 in hepatocellular carcinoma (HCC). AC092171.4 was significantly upregulated in HCC tumor tissues compared to normal liver tissues. HCC patients with high AC092171.4 expression showed poorer overall survival (OS) and disease-free survival (DFS) than those with low AC092171.4 expression. *In vitro* cell proliferation, migration and invasiveness were all higher in AC092171.4-overexpressing HCC cells, but lower in AC092171.4-silenced HCC cells, than in controls. Balb/c nude mice injected with AC092171.4-silenced HCC cells had smaller xenograft tumors, which showed less growth and pulmonary metastasis than control tumors. Bioinformatics analyses and dual luciferase reporter assays confirmed that AC092171.4 binds directly to miR-1271, which targets the 3’UTR of GRB2 mRNA. AC092171.4 expression correlates negatively with miR1271 expression and correlates positively with GRB2 mRNA expression in HCC tissues from patients. HCC cells co-transfected with miR-1271 mimics and sh-AC092171.4 show less proliferation, migration, invasiveness, GRB2 protein, and epithelial to mesencyhmal transition (EMT) than sh-AC092171.4-transfected HCC cells. These findings demonstrate that AC092171.4 promotes growth and progression of HCC by sponging miR-1271 and upregulating GRB2. This makes AC092171.4 a potential prognostic indicator and therapeutic target for HCC patients.

## INTRODUCTION

Hepatocellular carcinoma (HCC) is the most common primary liver cancer and the second leading cause of cancer-related deaths worldwide [1]. The prognosis of HCC patients is poor despite the availability of several treatment options because of high recurrence rates and diagnosis in advanced stages [2]. Hence, there is an urgent need to discover new diagnostic markers and therapeutic targets to improve the prognosis of HCC patients.

Long noncoding RNAs (lncRNAs) are valuable biomarkers and potential therapeutic targets in several cancers [3, 4]. LncRNAs are noncoding RNAs that lack an open reading frame and are >200 nucleotides long [5]. Xu et al reported that lncRNA Myd88 promotes HCC growth and metastasis by regulating Myd88 expression through H3K27 modifications [6]. Liao et al showed that lncRNA AC092171.4 expression correlates with poor prognosis in HCC patients [7]. However, the mechanism through which AC092171.4 regulates HCC growth and progression is not clear. Therefore, in this study, we investigated the role of lncRNA AC092171.4 in HCC growth and progression, and its mechanism of action.

## RESULTS

### High AC092171.4 expression correlates with poor prognosis in HCC patients

**Figure 1:**
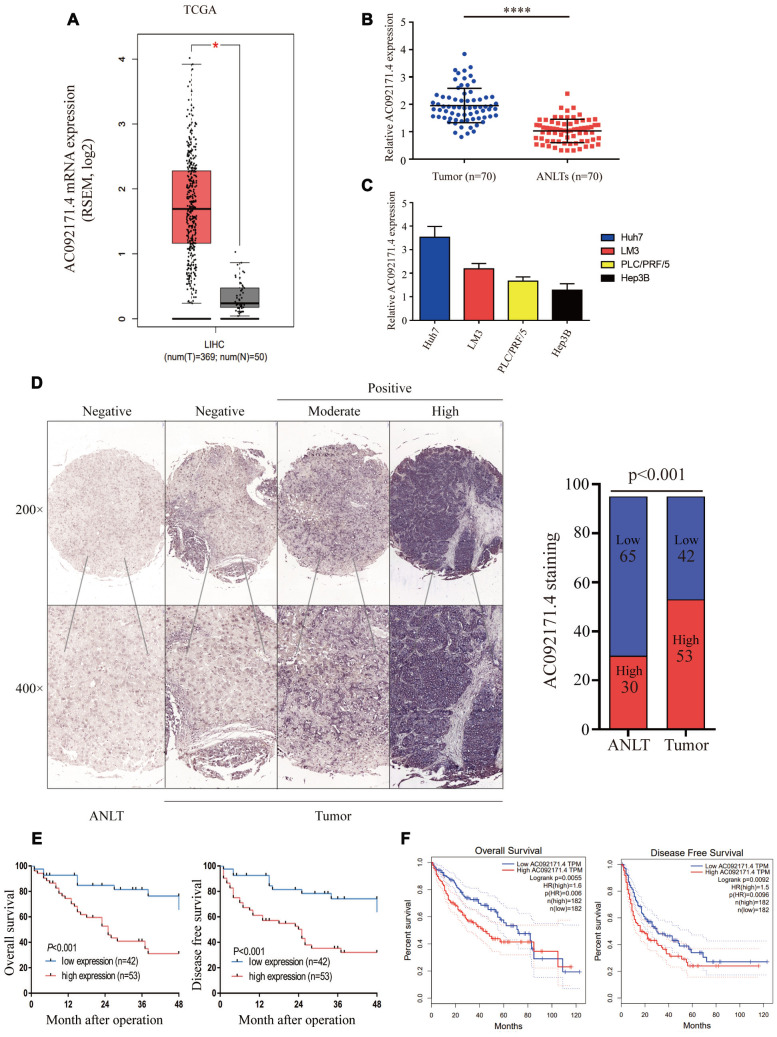
**AC092171.4 is upregulated in HCC tissues.** (**A**) Quantitative real-time PCR (qRT-PCR) analysis of AC092171.4 expression in HCC (n=369) and normal liver (n=50) tissues from the GEPIA database. As shown, AC092171.4 levels are higher in HCC tissues compared to normal liver tissues (^*^p<0.05). (**B**) QRT-PCR analysis of AC092171.4 expression in 70 pairs of HCC and corresponding ANLTs. As shown, AC092171.4 levels are higher in HCC tissues compared to adjacent normal liver tissues (ANLTs; ^****^p<0.0001). (**C**) QRT-PCR analysis showing AC092171.4 expression was significantly higher in the Huh7 and LM3 HCC cell lines than PLC/PRF/5 and Hep3B cells. (**D**) Chromogenic *in situ* hybridization (CISH) analysis of AC092171.4 expression in 95 pairs of HCC and ANLTs in a representative photograph. The results show that AC092171.4 expression is higher in HCC tissues compared to ANLTs (*p<0.001*). (**E**) Kaplan-Meier survival curve analyses of overall survival (OS) and disease-free survival (DFS) in HCC patients with low (n=42) and high (n=53) AC092171.4 expression. (**F**) Kaplan-Meier survival curve analyses of overall survival (OS) and disease-free survival (DFS) in high and low AC092171.4-expressing HCC patients of the GEPIA dataset (tumor=369, normal=50). * denotes p<0.05.

We analyzed AC092171.4 expression in HCC (n=369) and normal liver tissues (n=50) in the TCGA datasets. AC092171.4 expression was significantly higher in the HCC tissues compared to normal liver tissues (Figure 1A; *p<0.05*). Furthermore, we analyzed AC092171.4 expression in 70 pairs fresh HCC and the corresponding adjacent normal liver tissues (ANLTs). Quantitative real-time PCR (qRT-PCR) analysis showed that AC092171.4 expression was significantly higher in the HCC tissues compared to the ANLTs (*p<0.0001*; Figure 1B). QRT-PCR analysis also showed that AC092171.4 expression was higher in the Huh7 and LM3 HCC cell lines than PLC/PRF/5 and Hep3B cells (Figure 1C). CISH analysis in 95 paired HCC and ANLTs confirmed that AC092171.4 levels were significantly higher in HCC tissues compared to the ANLTs (*p<0.001*; Figure 1D). Furthermore, AC092171.4 expression was significantly associated with age (*p=0.048*), α-fetoprotein (AFP) levels (*p<0.001*), tumor size (*p<0.001*), the number of tumor nodules (*p=0.007*), and the presence of cancer emboli (*p=0.005*). (Table 1 and Supplementary Table 1)

**Table 1:** Correlation between AC092171.4 expression with clinicopathological characteristics of HCC.

**Clinicopathological Variables**	**n**	**AC092171.4 Expression**		***P* Value**
		**Low (42)**	**High (53)**	
Sex				
Male	81	33	48	0.101
Female	14	9	5	
Age, years				
<50	48	26	22	**0.048**
≥50	47	16	31	
AFP, ng/L				
<200	50	31	19	**0.000**
≥200	45	11	34	
HBsAg				
Negative	41	19	22	0.716
Positive	54	23	31	
Tumor size, cm			
≤5	49	31	18	**0.000**
>5	46	11	35	
Tumor nodule number			
Solitary	58	32	26	**0.007**
Multiple (≥2)	37	10	27	
Cancer embolus			
Absence	65	35	30	**0.005**
Presence	30	7	23	
TNM stage				
Early (I & II)	51	26	28	
Late (III & IV)	44	16	28	0.153
Differentiation grade			
Well	64	31	33	
Poor	31	11	20	0.233

Abbreviation: AFP, alpha fetoprotein; HBsAg, hepatitis B surface antigen.

### AC092171.4 expression correlates with prognosis of HCC patients

Next, we used Kaplan-Meier survival curves to analyze the prognostic value of AC092171.4 expression in HCC patients. In CISH analysis, HCC patients with high AC092171.4 expression showed worse OS and DFS than HCC patients with low AC092171.4 expression (*p<0.001*; Figure 1E). In the GEPIA dataset, HCC patients with high AC092171.4 expression were associated with worse OS (*p=0.006*) and DFS (*p=0.0096*) rates compared to those with low AC092171.4 expression (Figure 1F). Multivariate analysis showed that AC092171.4 expression is an independent predictor of OS and DFS in HCC patients (*p=0.02* and *p=0.03,* respectively; Tables 2 and 3). Overall, high AC092171.4 expression predicts poor prognosis in HCC patients.

**Table 2:** Univariate and multivariate Cox regression analysis of risk factors associated with overall survival.

**Variables**	**Univariate analysis**				**Multivariate analysis**		
	**HR**	**95% CI**	***P* Value**		**HR**	**95% CI**	***P* Value**
AC092171.4 expression (High vs. Low)	3.35	1.64-6.84	**<0.01**		2.66	1.14-6.19	**0.02**
Sex (Male vs. Female)	0.34	0.11-1.12	0.08				
Age (≥50 vs. <50)	1.10	0.60-2.01	0.76				
HBsAg (Positive vs. Negative)	1.42	0.76-2.68	0.27				
AFP (≥200 ng/ml vs. <200ng/ml)	2.18	1.17-4.06	**0.02**		1.05	0.50-2.21	0.91
Tumor size (>5 cm vs. ≤5 cm)	2.55	1.33-4.92	**<0.01**		1.36	0.67-2.77	0.39
Tumor nodule number (Multiple vs. Single)	1.85	1.01-3.39	**<0.05**		0.81	0.39-1.71	0.58
Cancer embolus (Presence vs. Absence)	2.93	1.59-5.38	**<0.01**		1.93	0.91-4.08	0.08
TNM stage (Late vs. Early)	2.60	1.39-4.86	**<0.01**		2.00	0.99-4.04	0.05
Differentiation grade (Poor vs. Well)	1.87	1.02-3.44	**<0.05**		1.25	0.65-2.39	0.51

Abbreviation: AFP, alpha fetoprotein; HBsAg, hepatitis B surface antigen.

**Table 3:** Univariate and multivariate Cox regression analysis of risk factors associated with disease-free survival.

**Variables**	**Univariate analysis**				**Multivariate analysis**		
	**HR**	**95% CI**	***P* Value**		**HR**	**95% CI**	***P* Value**
AC092171.4 expression (High vs. Low)	3.35	1.71-7.13	**<0.01**		2.51	1.08-5.84	**0.03**
Sex (Male vs. Female)	0.48	0.17-1.34	0.16				
Age (≥50 vs. <50)	1.02	0.56-1.88	0.94				
HBsAg (Positive vs. Negative)	1.49	0.79-2.80	0.22				
AFP (≥200 ng/ml vs. <200ng/ml)	2.29	1.23-4.27	**<0.01**		0.99	0.46-2.14	0.98
Tumor size (>5 cm vs. ≤5 cm)	3.42	1.72-6.81	**<0.01**		1.99	0.96-4.12	0.06
Tumor nodule number (Multiple vs. Single)	2.19	1.19-4.01	**0.01**		1.14	0.56-2.33	0.71
Cancer embolus (Presence vs. Absence)	2.78	1.50-5.11	**<0.01**		1.41	0.69-2.87	0.35
TNM stage (Late vs. Early)	2.92	1.55-5.51	**<0.01**		2.27	1.12-4.63	**0.02**
Differentiation grade (Poor vs. Well)	1.87	1.02-3.44	**0.04**		1.08	0.54-2.16	0.83

Abbreviation: AFP, alpha fetoprotein; HBsAg, hepatitis B surface antigen.

### AC092171.4 silencing inhibits *in vitro* proliferation, migration and invasion of HCC cells.

**Figure 2:**
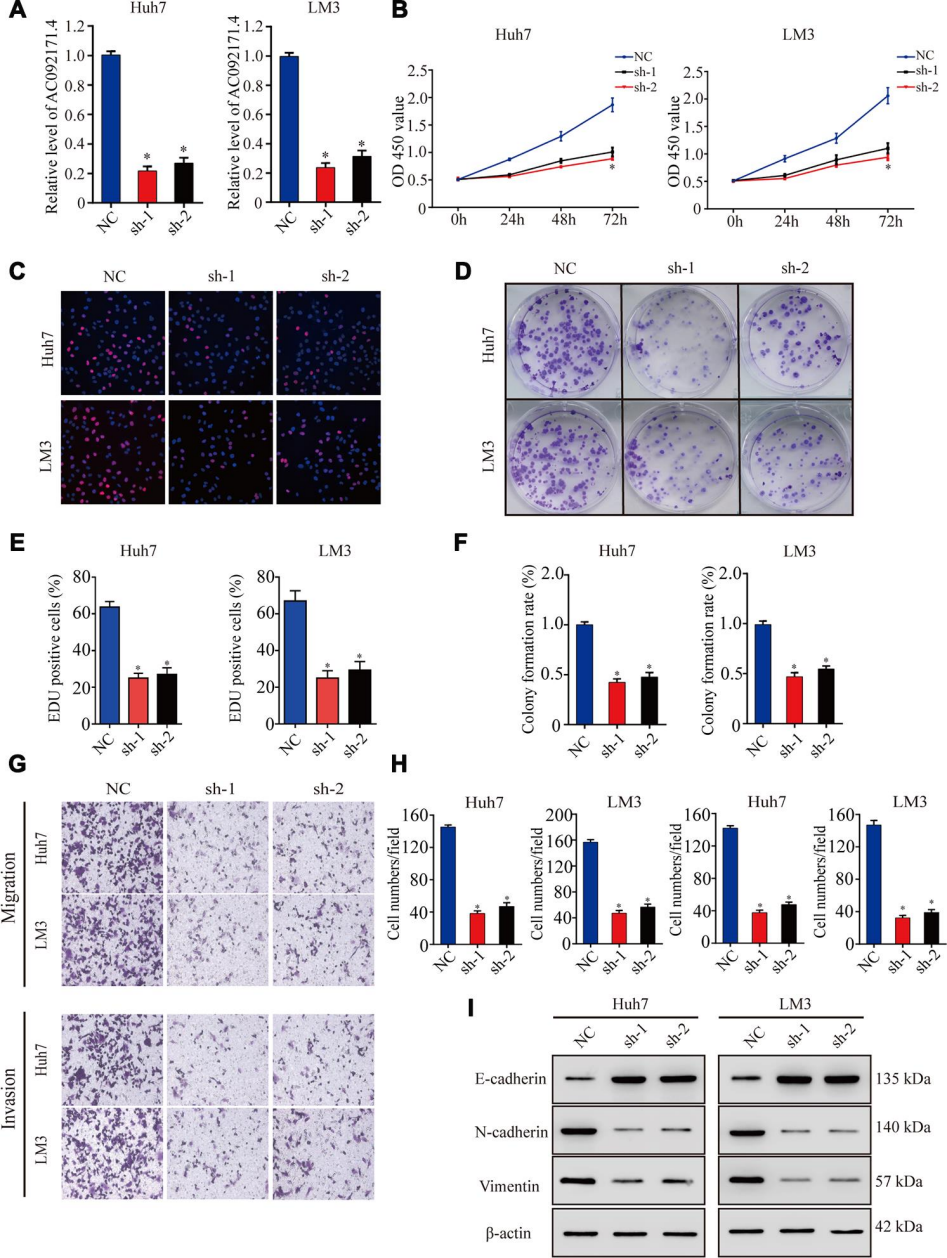
**AC092171.4 silencing decreases proliferation, migration and invasion in HCC cell lines.** (**A**) QRT-PCR analysis shows AC092171.4 expression in the sh-NC and sh-AC092171.4 transfected Huh7 and LM3 cells. (**B**) CCK-8 assay results show proliferation status of the sh-NC and sh-AC092171.4 transfected Huh7 and LM3 cells. (**C**) EdU assay results show proliferation status of the sh-NC and sh-AC092171.4 transfected Huh7 cells. (**D**) Colony formation assay results show the total number of colonies in the sh-NC and sh-AC092171.4 transfected Huh7 cells. (**E**) EdU assay results show proliferation status of the sh-NC and sh-AC092171.4 transfected LM3 cells. (**F**) Colony formation assay results show the total number of colonies in the sh-NC and sh-AC092171.4 transfected LM3 cells. (**G**) Transwell migration assay results show AC092171.4 downregulation inhibited cell migration and invasion after transfection. (**H**) Transwell invasion assay results show the numbers of invasive sh-NC and sh-AC092171.4 transfected Huh7 and LM3 cells. (**I**) Representative western blot shows the expression of E-cadherin (epithelial marker) as well as N-cadherin and vimentin (mesenchymal markers) in the sh-NC and sh-AC092171.4 transfected Huh7 and LM3 cells. β-actin was used as loading control. * denotes p<0.05.

As shown in Figure 2A, AC092171.4 levels were significantly reduced in sh-AC092171.4 transfected Huh7 and LM3 cells compared to controls (*p<0.05*). CCK-8, EdU and colony formation assays showed that cell proliferation was significantly reduced in AC092171.4 knockdown Huh7 and LM3 cells compared to controls (Figure 2B–2F). Transwell assays showed that migration and invasiveness of AC092171.4 silenced HCC cells were significantly reduced compared to the corresponding controls (Figure 2G, 2H). These data demonstrate that AC092171.4 knockdown inhibits *in vitro* proliferation, migration and invasiveness of HCC cells.

### AC092171.4 overexpression enhances proliferation, invasion, and migration of HCC cells

**Figure 3:**
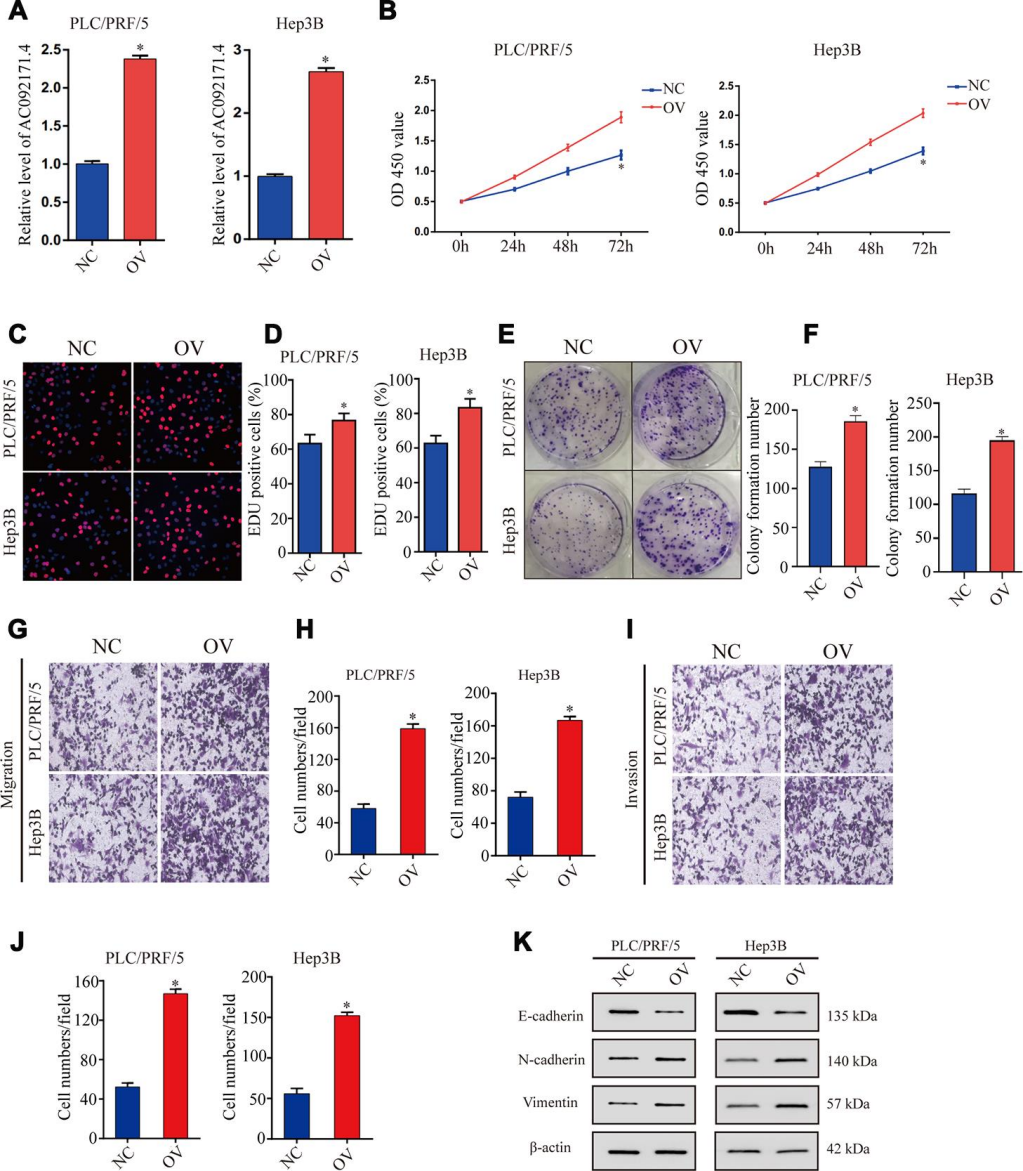
**AC092171.4 overexpression increases proliferation, invasion and migration in HCC cell lines.** (**A**) QRT-PCR analysis shows AC092171.4 levels in the control and AC092171.4 overexpressing PLC/PRF/5 and Hep3B cells. (**B**) CCK-8 assay results show cell proliferation status of control and AC092171.4 overexpressing PLC/PRF/5 and Hep3B cells. (**C**) EdU assay results show proliferation status of control and AC092171.4 overexpressing PLC/PRF/5 cells. (**D**) EdU assay results show proliferation status of control and AC092171.4 overexpressing Hep3B cells. (**E**) Colony formation assay results show the total number of colonies in control and AC092171.4 overexpressing PLC/PRF/5 cells. (**F**) Colony formation assay results show the total number of colonies in control and AC092171.4 overexpressing Hep3B cells. (**G** and **H**) Transwell migration assay results show the total numbers of migrating control and AC092171.4 overexpressing PLC/PRF/5 and Hep3B cells. (**I** and **J**) Transwell invasion assay results show the total numbers of invasive control and AC092171.4 overexpressing PLC/PRF/5 and Hep3B cells. (**K**) Representative western blot shows the expression of E-cadherin (epithelial marker) as well as N-cadherin and vimentin (mesenchymal markers) in control and AC092171.4 overexpressing PLC/PRF/5 and Hep3B cells. * denotes p<0.05.

As shown in Figure 3A, AC092171.4 levels were significantly higher in AC092171.4-overexpressing PLC/PRF/5 and Hep3B cells compared to the corresponding controls. CCK-8, EdU and colony formation assays showed that cell proliferation was significantly higher in AC092171.4-overexpressing PLC/PRF/5 and Hep3B cells compared to their corresponding controls (Figure 3B–3F). Furthermore, Transwell assays showed that migration and invasiveness of AC092171.4 overexpressing HCC cells was significantly enhanced compared to their corresponding controls (Figure 3E and 3J). These data suggest that AC092171.4 promotes *in vitro* proliferation, migration, and invasion of HCC cells.

### Tumor growth and pulmonary metastasis is reduced in nude mice xenografted with AC092171.4-silenced HCC cells

**Figure 4:**
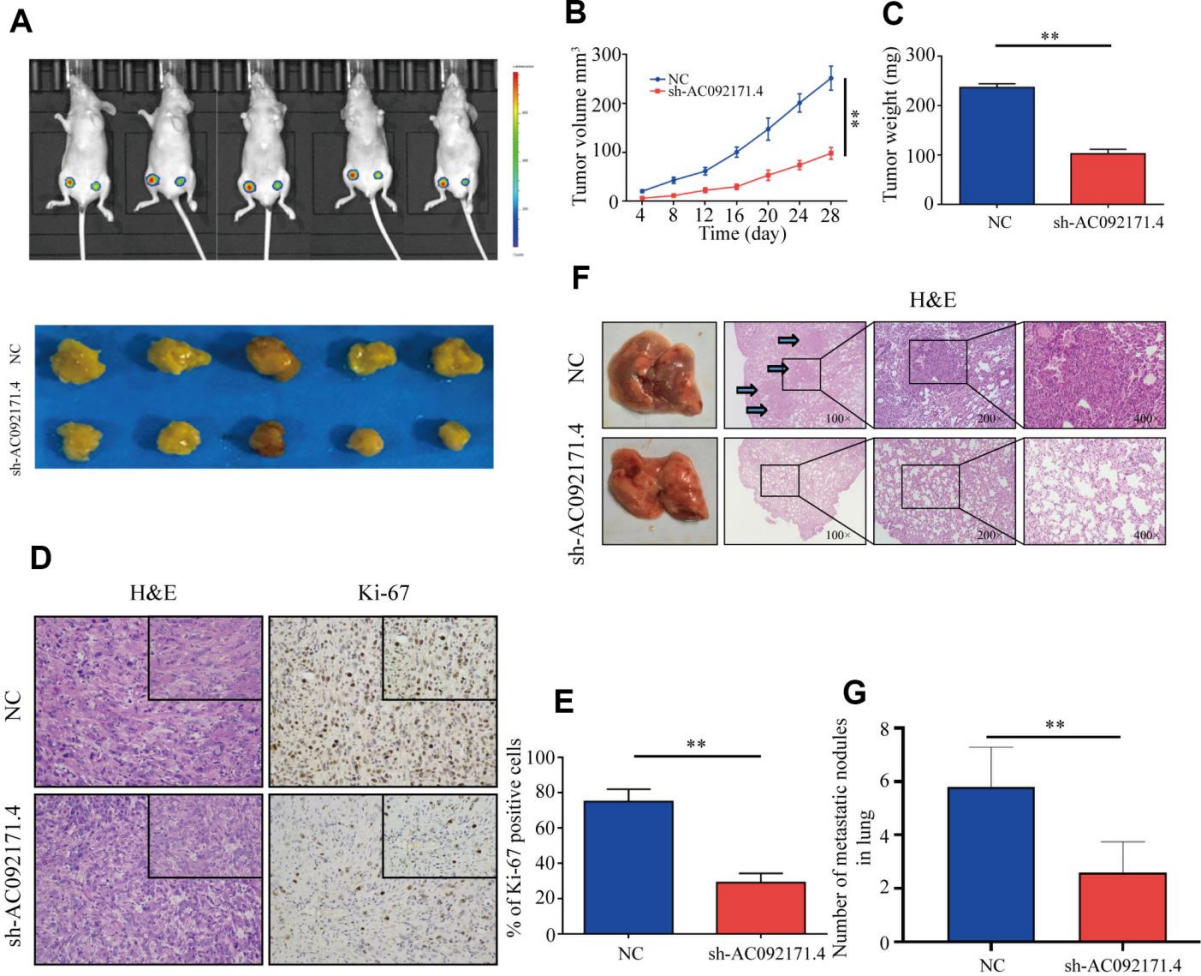
**AC092171.4 knockdown represses *in vivo* xenograft HCC tumor growth and pulmonary metastases.** (**A**) Balb/c nude mice were subcutaneously injected with sh-NC or sh-AC092171.4 transfected Huh7 cells and tumor from respective groups were shown (n=5). (**B**, **C**) Xenograft tumor volume and weights in nude mice subcutaneously injected with sh-NC or sh-AC092171.4 transfected Huh7 cells. (**D**, **E**) Immunohistochemical analysis shows percentage of Ki-67-positive stained cells in the sections of xenograft tumors from control and AC092171.4 knockdown nude mice. (**F**, **G**) Total numbers of pulmonary metastatic nodules in nude mice injected with control and AC092171.4 knockdown Huh-7 cells through the tail vein. * denotes p<0.05.

Next, we analyzed xenograft HCC tumor growth by subcutaneously injecting control and AC092171.4-knockdown Huh7 cells into Balb/c nude mice. As shown in Figure 4A, tumor growth was significantly reduced in AC092171.4-knockdown group mice compared to the control group mice. Tumor volume and weight in the AC092171.4 knockdown group mice was significantly reduced at 4 weeks compared to the control group mice (Figure 4B, 4C). Furthermore, we observed significant reduction in Ki-67-positive cells in xenograft tumors from the AC092171.4-knockdown group mice compared to the control group mice (Figure 4D, 4E). Next, we analyzed pulmonary metastasis by injecting control or AC092171.4-knockdown HCC cells into the tail vein of Balb/c nude mice. The total numbers of pulmonary metastatic nodules were significantly lower in the AC092171.4-knockdown group mice compared to the control group mice (Figure 4F). Moreover, H&E staining of lung sections demonstrated that the metastatic nodule size was significantly reduced in the AC092171.4 knockdown group mice compared to the control group mice (Figure 4G). These results demonstrate that AC092171.4 promotes *in vivo* growth and metastasis of HCC cells.

### AC092171.4 regulates growth factor receptor-bound 2 (GRB2) protein levels by competitively binding miR-1271

**Figure 5:**
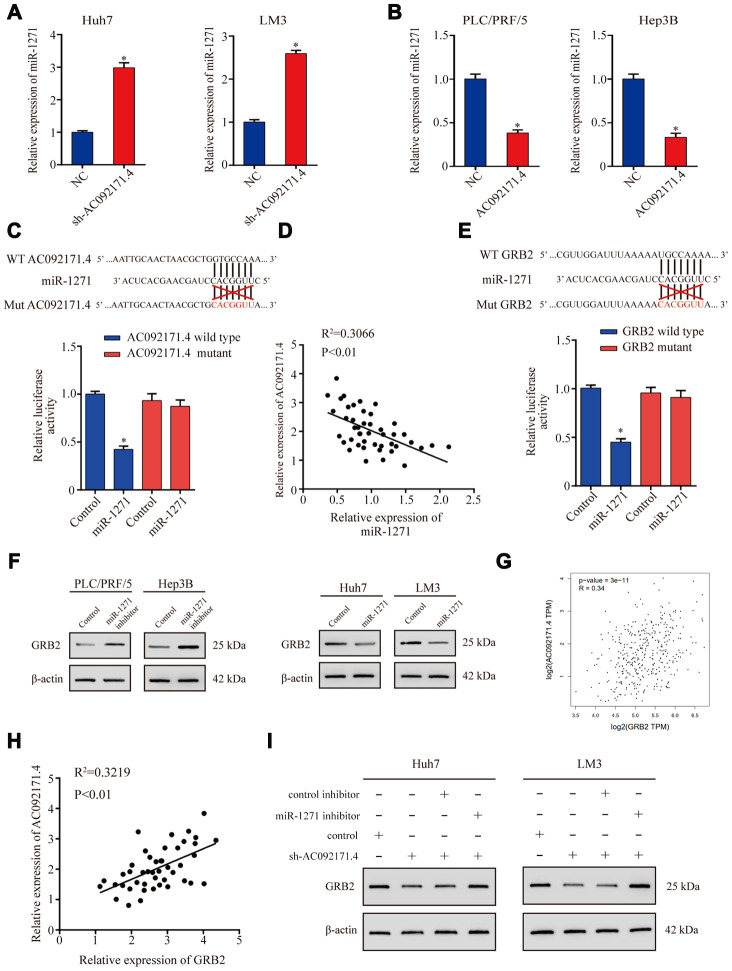
**AC092171.4 regulates GRB2 protein expression by competitively binding miR-1271.** (**A**) QRT-PCR analysis shows miR-1271 levels in sh-NC and sh-AC092171.4-transfected HCC cells. (**B**) QRT-PCR analysis shows miR-1271 levels in control and AC092171.4 overexpressing HCC cells. (**C**) Dual luciferase reporter assay results show relative firefly luciferase activity in HCC cells transfected with wild-type or mutant AC092171.4 and miR-1271 mimics. (**D**) Pearson’s correlation analysis shows the association between AC092171.4 and miR-1271 levels in 45 HCC tissue samples from 70 pair HCC specimens. (**E**) Dual luciferase reporter assay results show relative firefly luciferase activity in HCC cells transfected with WT or mutant 3’UTR of GRB2 and miR-1271 mimics. (**F**) Western blot results show GRB2 protein levels in HCC cells transfected with miR-1271 mimics or miR-1271 inhibitors. (**G**) Pearson’s correlation analysis shows the relationship between AC092171.4 and GRB2 mRNA levels in HCC tissues from the TCGA datasets in GEPIA website. (**H**) Pearson’s correlation analysis shows the relationship between AC092171.4 and GRB2 mRNA levels in HCC tissues from the TCGA datasets. (**I**) Western blot analysis shows GRB2 protein expression in HCC cells transfected with AC092171.4-shRNA plus miR-1271 inhibitor or AC092171.4-shRNA alone. * denotes p<0.05.

LncRNAs modulate tumor progression by competitively binding miRNAs [4, 8]. Therefore, we used miRDB and miRcode softwares and identified a potential binding site for miR-1271 in the AC092171.4 sequence. QRT-PCR analysis showed that miR-1271 expression was significantly higher in AC092171.4 knockdown HCC cells (Figure 5A). Conversely, miR-1271 levels were significantly reduced in AC092171.4 overexpressing HCC cells (Figure 5B). Furthermore, dual luciferase reporter assay results demonstrated that reporter firefly luciferase activity was significantly reduced in HCC cells co-transfected with vector containing wild-type (WT) AC092171.4 and miR-1271 mimics compared to cells co-transfected with vector containing mutant AC092171.4 and miR-1271 mimics (Figure 5C). Pearson’s correlation analysis of 45 HCC tissue specimens showed that AC092171.4 expression negatively correlated with miR-1271 expression (Figure 5D). These results show that the AC092171.4 regulates growth and progression of HCC through miR-1271.

Next, we investigated the genes targeted by miR-1271 using TargetScan Human 7.1 and miRDB softwares and found a potential miR-1271 binding site in the GRB2 3’-UTR. Dual luciferase reporter assay results showed that firefly luciferase reporter activity was significantly reduced in HCC cells co-transfected with pCMV-GRB2-WT vector and miR-1271 mimics compared to cells co-transfected with pCMV-GRB2-Mut vector and miR-1271 mimics (Figure 5E). Western blot analysis showed that GRB2 protein levels were significantly higher in miR-1271 knockdown HCC cells and significantly reduced in miR-1271 overexpressing HCC cells (Figure 5F). Pearson’s correlation analysis showed that AC092171.4 expression positively correlated with GRB2 mRNA expression in the HCC specimens from the TCGA datasets [9] and the 45 HCC tissue specimens (Figure 5G and 5H). Western blot analysis showed that GRB2 expression was significantly reduced in sh-AC092171.4 transfected HCC cells, but was higher in HCC cells co-transfected with sh-AC092171.4 and miR-1271 inhibitor (Figure 5I). These results show that AC092171.4 enhances GRB2 expression by competitively sponging miR-1271.

### AC092171.4 promotes growth and progression of HCC cells by regulating GRB2 protein expression via miR-1271

**Figure 6:**
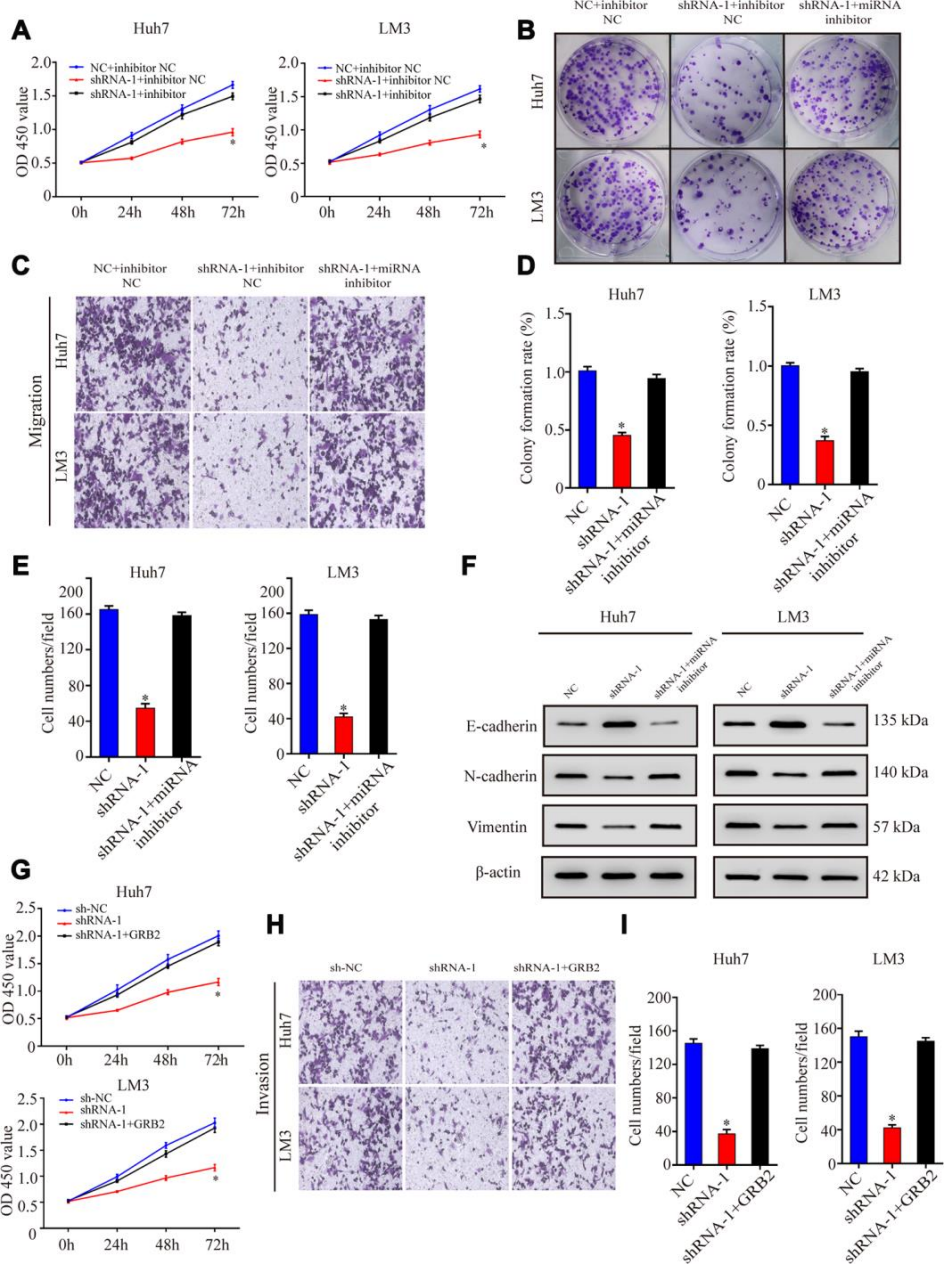
**AC092171.4 promotes proliferation, migration, and invasiveness of HCC cells by suppressing miR-1271-dependent downregulation of GRB2 protein translation.** (**A**) CCK-8 assay results show proliferation of Huh-7 and LM3 cells transfected with sh-NC, sh-AC092171.4, sh-NC plus miR-1271 inhibitor, or sh-AC092171.4 plus miR-1271 inhibitor. (**B**) Colony formation assays show the numbers of colonies formed by Huh-7 cells transfected with sh-NC, sh-AC092171.4, sh-NC plus miR-1271 inhibitor, or sh-AC092171.4 plus miR-1271 inhibitor. (**C**) Transwell assay results show the total numbers of migratory and invasive Huh-7 cells transfected with sh-NC, sh-AC092171.4, sh-NC plus miR-1271 inhibitor, or sh-AC092171.4 plus miR-1271 inhibitor. (**D**) Colony formation assays show the numbers of colonies formed by LM3 cells transfected with sh-NC, sh-AC092171.4, sh-NC plus miR-1271 inhibitor, or sh-AC092171.4 plus miR-1271 inhibitor. (**E**) Transwell assay results show the total numbers of migratory and invasive LM3 cells transfected with sh-NC, sh-AC092171.4, sh-NC plus miR-1271 inhibitor, or sh-AC092171.4 plus miR-1271 inhibitor. (**F**) Western blot analysis shows relative levels of E-cadherin, N-cadherin and vimentin levels in HCC cells transfected with shRNA-AC092171.4 alone or shRNA-AC092171.4 plus miR-1271 inhibitor. (**G**) CCK-8 assay results show the proliferation status in AC092171.4-silenced and sh-AC092171.4-silenced plus GRB2 overexpressing Huh7and LM3 cells. (**H**, **I**) Transwell assay results show the migration status of AC092171.4-silenced and sh-AC092171.4-silenced plus GRB2 overexpressing Huh7and LM3 cells. * denotes p<0.05.

Next, we investigated if AC092171.4 promotes HCC growth by regulating GRB2 expression via miR-1271. CCK-8 assay showed that proliferation of HCC cells co-transfected with sh-AC092171.4 plus miR-1271 inhibitor was significantly higher than those transfected with sh-AC092171.4 alone, but comparatively lower than HCC cells transfected with sh-NC and sh-NC plus miR-1271 inhibitor (Figure 6A). Furthermore, colony formation and Transwell assays showed that growth, migration, and invasiveness of HCC cells co-transfected with sh-AC092171.4 plus miR-1271 inhibitor were significantly higher than the sh-AC092171.4-transfected HCC cells, but comparatively lower than HCC cells transfected with sh-NC and sh-NC plus miR-1271 inhibitor (Figure 6B–6E). Moreover, transient transfection of GRB2 partially enhances the proliferation, growth, migration, and invasiveness of sh-AC092171.4-transfected HCC cells compared to sh-AC092171.4-transfected HCC cells alone (Figure 6G–6I).

### AC092171.4 enhances EMT in HCC cells by competitively binding miR-1271

Next, we analyzed EMT status of HCC cells with differential AC092171.4 expression by analyzing specific EMT protein markers by western blotting. AC092171.4-knockdown Huh7 and LM3 cells showed significantly higher E-cadherin (epithelial marker) protein levels and reduced N-cadherin and vimentin (mesenchymal markers) protein levels compared to the corresponding controls (Figure 2I). Conversely, AC092171.4-knockdown Huh7 and LM3 cells showed significantly higher N-cadherin and vimentin protein levels and reduced E-cadherin protein levels compared to the corresponding controls (Figure 3K). Furthermore, HCC cells transfected with shRNA-AC092171.4 showed increased expression of E-cadherin and decreased expression of N-cadherin and vimentin compared to HCC cells transfected with sh-NC, and partially reversed by miR-1271 inhibitor (Figure 6F). In summary, our study suggests that AC092171.4 regulates Grb2-dependent HCC growth and progression by inhibiting miR-1271.

## DISCUSSION

LncRNAs have emerged as important regulators of tumor cell growth and progression in several cancers [10]. Our study demonstrates that high AC092171.4 expression is associated with worse survival outcomes in HCC patients. We also demonstrate that AC092171.4 promotes *in vitro* and *in vivo* growth and progression of HCC using cell lines and xenograft tumor nude mice models.

Many studies have demonstrated that lncRNA regulate tumor cell growth and survival by acting as “sponges” for specific miRNA [11, 12]. For example, lncXIST regulates lung cancer growth and progression by sponging miR-335 [13]. Previous studies have shown that miR1271 acts as a tumor suppressor in HCC [14, 15]. The results of the dual luciferase reporter assay confirmed that miR-1271 is a direct target of AC092171.4. Furthermore, we demonstrate that miR-1271 overexpression suppresses the oncogenic function of AC092171.4 in HCC cell lines.

The miRNAs bind to the 3’-UTR sequences of the target mRNAs and inhibit protein translation [16]. Dual luciferase reporter assay confirmed that GRB2 is a direct target of miR-1271. GRB2 is a widely expressed adapter protein composed of an intermediate SH2 (Src homology 2) domain and two SH3 (Src homology 3) domains [17, 18]. GRB2 is upregulated in non-small cell lung cancer and colorectal cancers and regulates tumor progression [19, 20]. We demonstrate that AC092171.4 knockdown decreases GRB2 protein expression in HCC cells. Furthermore, transient GRB2 expression restores growth, proliferation, and progression of AC092171.4 knockdown HCC cells. Moreover, GRB2 expression is increased by transfecting AC092171.4 knockdown HCC cells with the miR-1271 inhibitor. Previous studies show that GRB2 promotes tumorigenesis by binding to Sos1 or its adaptor protein, Gab1 [21, 22]. Moreover, GRB2 promotes EMT in HCC via ERK/AKT signaling pathway [23]. EMT drives migration and invasion of HCC cells [24, 25]. We demonstrate that knockdown of AC092171.4 increases the levels of E-cadherin, an epithelial cell marker, and decrease the levels of mesenchymal cell markers, vimentin and N-cadherin. However, treatment with miR-1271 inhibitor reverses these effects in the AC092171.4-kncokdown HCC cells.

In summary, our study demonstrates that lncRNA AC092171.4 promotes GRB2-dependent HCC progression by competitively binding miR-1271. Our study also suggests that AC092171.4 is a potential prognostic indicator and a therapeutic target in HCC.

## METHODS

### HCC patient specimens

We obtained HCC and adjacent normal liver tissue (ANLT; normal liver tissue 3 cm away from the HCC tumor) samples from 95 cases HCC patients who underwent hepatectomy at the First Affiliated Hospital of Sun Yat-Sen University (Guangzhou, China) between July 2013 and December 2014 and 70 cases in 2018. Patients who received radiotherapy or chemotherapy before surgery were excluded from the study [26]. The clinical characteristics of all patients whose specimens were confirmed by a pathologist are shown in Table 1 and Supplementary Table 1. The HCC and ANLT specimens were stored in RNAlater solution (Invitrogen, USA), frozen in liquid nitrogen, and stored at -80 °C immediately after resection. Overall survival (OS) time is defined as the period between the date of surgery and the date of death or date of last follow-up. Disease-free survival (DFS) time is defined as the period between the date of surgery and the date of cancer recurrence until June 2018. This study was approved by the Ethics Committee of the First Affiliated Hospital of Sun Yat-Sen University. The study conformed to the 1964 Declaration of Helsinki and its later amendments or comparable ethical standards. We obtained written informed consent from all patients included in this study.

### HCC cell lines

The human HCC cell lines (Huh7, LM3, PLC/PRF/5 and Hep3B) were obtained from the Institute of Biochemistry and Cell Biology (Chinese Academy of Sciences, Shanghai, China) and stored in liquid nitrogen. The HCC cell lines were cultured in a humidified incubator at 37 °C and 5% CO_2_ in DMEM medium (Gibco, USA) supplemented with 10% fetal bovine serum (FBS), 100 μg/mL streptomycin and 100 U/mL penicillin (Sigma, USA).

### Gene expression profiling interactive analysis (GEPIA) dataset

The lncRNA expression data and the corresponding clinical information of the HCC patients, HCC (n=369) and normal liver tissues (n=50), were obtained from GEPIA (http://gepia.cancer-pku.cn/), a public database that is available for analyzing the gene expression microarray data from the TCGA projects [9].

### Quantitative real-time polymerase chain reaction (qRT-PCR)

We extracted total RNA from HCC and ANLT tissues, and HCC cell lines using TRIzol (Invitrogen, NY, USA) according to the manufacturer’s instructions. QRT-PCR was performed using the SYBR green detection RT-PCR system (Takara, Japan) according to manufacturer’s instructions. The following primers were obtained from Servicebio Technology (Wuhan, China): AC092171.4 forward: 5’-ATTACCCCGCCCTGGATTTG-3’, reverse: 5’-TTGTTTTCCCCACCCC-3’; miR-1271 forward: 5’-CAGCACTTGGCACCTAGCA-3’, reverse: 5’-TATGGTTGTTCTCCTCTCTGTCTC-3’; GRB2 forward: 5’-GGGCCTTTCTTATCCGAGA-3’, reverse: 5’-TGCACATCGTTTCCAAACTT-3’; β-actin forward: 5’-CACCCAGCACAATGAAGATCAAGAT-3’, reverse: 5’-CCAGTTTTTAAATCCTGAGTCAAGC-3’; U6 forward: 5’-CTCGCTTCGGCAGCACA-3’, reverse: 5’-AAACGCTTCACGAATTTGCGT-3’. β-actin and U6 were used as internal controls. All samples were analyzed in triplicate. The relative mRNA, lncRNA, and miRNA expressions were determined using the 2^-ΔΔCt^ method. (Supplementary Figure 1)

### Chromogenic *in situ* hybridization (CISH) analysis

We performed chromogenic in situ hybridization (CISH) analysis to determine AC092171.4 expression in 95 pairs of HCC tumor tissues and ANLTs. The AC092171.4 probe for CISH was 5'- CTCCCTCAAATCAGGATGGG -3'. In brief, the tissues were fixed in 4% paraformaldehyde (DEPC, Servicebio) for 2.5 h followed by incubation in prehybridization buffer for 1 h. Then, the samples were incubated in fresh hybridization buffer containing 8 ng/ml of the digoxigenin-labeled probe for 24 h. Finally, the tissues were mounted on slides in neutral balsam (Sinopharm Chemical Reagent Co., Ltd) and photo-graphed using a bright field microscope (Leica, Germany).

### Cell transfections

We obtained lentiviral vectors with shRNAs against AC092171.4 from Servicebio (Guangzhou, China), and miR-1271 inhibitors and miR-1271 mimics from RiboBio (Guangzhou, China). The AC092171.4 and GRB2 CDS sequences were cloned into the pCMV expression vector (Invitrogen) for overexpression. The transfections were performed using Lipofectamine 2000 (Invitrogen, Grand Island, NY, USA) according to the manufacturer’s instructions. Stable AC092171.4 knockdown HCC cell lines were obtained by incubating the cells with lentiviruses and 8 mg/ml polybrene (Sigma, USA) for 72 h followed by culturing in selection media containing 2 mg/ml puromycin for 3 days.

### Dual luciferase reporter assays

We used bioinformatics tools such as TargetScan Human 7.1 (http://www.targetscan.org/vert_72/) and miRDB (http://mirdb.org/miRDB/index.html) were used to identify miR-1271 binding sites in the AC092171.4 sequence and miR-1271 binding sites in the GRB2 sequence. We prepared firefly luciferase containing plasmid vectors (psiCHECK^TM^-2, Servicebio) containing wild-type (WT) or mutant (Mut) 3’UTR sequence of AC092171.4 (5’-AATTGCAACTAACGCTGCACGGUUA-3’) and wild-type (WT) or mutant (Mut) 3’UTR sequence of GRB2 (5’-CGUUGGAUUUAAAAACACGGUUA-3’). We co-transfected 293T with different combinations of plasmid vectors with WT or Mut constructs of AC092171.4 and GBR2-3’UTR along with miR-1271 mimics or negative control (NC) using Lipofectamine 2000 (Invitrogen, Carlsbad, CA) according to manufacturer’s instructions.

### Western blotting

We lysed the HCC cells and patient tissues using cold RIPA buffer containing protease inhibitors (1:100) and protein inhibitors (1:100). Then, the protein concentration in all samples was estimated using the BCA assay. Equal amounts of protein samples were separated on a 10% SDS-PAGE gel and transferred onto a nitrocellulose membrane. The membranes were blocked using 5% skimmed milk for 1 h. Then, the membranes were incubated overnight at 4^o^C with primary antibodies against GRB2 (1:1000; ab32037, Abcam), E-cadherin (1:1000; #3195, CST), N-cadherin (1:1000; #13116, CST), Vimentin (1:1000; #5741, CST) and *β*-actin (1:10000; ab6276, Abcam). Then, the membranes were incubated with the secondary HRP-conjugated antibody (KPL, USA). Then, the blots were developed using ECL (EMD Millipore, MA, USA). The ImageJ software was used to determine the relative expressions of different proteins using *β*-actin as the internal control.

### Cell counting kit-8 (CCK-8) assay

Cell Counting Kit-8 (CCK-8, Dojondo, Tabaru, Japan) assay was used to analyze cellular proliferation. We seeded 2×10^3^ cells into 96-well plates and added 10 μl of CCK-8 solution at 0, 24, 48, and 72 h for 2 h. Then, the optical density (OD) at 450 nm was measured at different time points using a microplate reader (Thermo-Fisher Scientific).

### Colony formation assay

We seeded 500 cells in a 6-well plate and let them grow for 12 days. Then, the colonies were fixed in 4% paraformaldehyde, stained with 1% crystal violet, and photographed and counted under a bright field light microscope.

### 5-Ethynyl-2’-deoxyuridine (EdU) assay

We incubated 5×10^3^ cells grown in 96-well plates with 50 μm EdU for 2 h. Then, we stained the cells with the DNA binding dye, 4’,6-diamidino-2-phenylindole (DAPI), and used a fluorescence microscope (Carl Zeiss AG, Germany) to determine the proportion of EdU-positive cells in each sample.

### Transwell migration and invasion assays

We used the Transwell Cell Migration Chambers and BiCoat Matrigel Invasion Chambers (Corning, NY, USA) to determine migration and invasion of HCC cells, respectively. We seeded 2×10^4^ HCC cells in 200 μL serum-free medium in the upper chamber and added 500 μL DMEM medium with 15% FBS in the lower chambers. After 24 h, we fixed the cells on the bottom chamber side of the cell membrane or the Matrigel with 4% paraformaldehyde, stained with 0.5% crystal violet, and photographed and counted the cells using a light microscope.

### Tumor xenograft model

The animal experiments were approved by the Animal Care and Use Committee of Sun Yat-Sen University. We divided four-week-old BLAB/c nude mice randomly into two groups (n=5), and subcutaneously injected 2×10^6^ control (NC) or AC092171.4 knockdown Huh7 cells. We measured tumor sizes every 4 days and monitored tumor progression using the Xenogen Spectrum small animal imaging system (Caliper, Hopkinton, MA). The mice were sacrificed at 4 weeks after injection. The tumor tissues were harvested and weighed. The tissues were then fixed in formaldehyde solution, sectioned, and subjected to immunohistochemistry using anti-Ki-67 antibodies (1:100; bs-2130R, Bioss, Beijing). To evaluate lung metastasis, we injected 1×10^6^ control (NC) or AC092171.4 knockdown Huh7 cells through the tail vein of nude mice. After six weeks, the mice were sacrificed, and the lung tissues were harvested and fixed in 4% formaldehyde solution. The tumors on the lung surface were counted. Hematoxylin-eosin (H&E) stained sections were evaluated to determine the extent of lung metastases.

### Statistical analysis

All data are represented as means ± SD. The χ2 test was used to analyze the relationships between categorical variables. The differences between groups were compared using the Student’s *t*-test. Kaplan-Meier survival curve and Cox regression analyses were used to analyze OS and DFS. Pearson’s correlation analyses was used to analyze the relationship of AC092171.4, miR-1271 and Grb2. *P* <0.05 was considered statistically significant. All statistical data was performed using the SPSS 20.0 statistical software.

## Supplementary Material

Supplementary Figure 1

Supplementary Table 1
