# Supplementary material for: Long noncoding RNA AC092171.4 promotes hepatocellular carcinoma progression by sponging microRNA-1271 and upregulating GRB2
Source: Aging (Albany NY). 2020 Jul 21;12(14):14141–56. doi: 10.18632/aging.103419 (PMC7425487; doi:10.18632/aging.103419)
Supplement: Supplementary Figure 1 [file aging-12-103419-s002..pdf]

SUPPLEMENTARY FIGURE

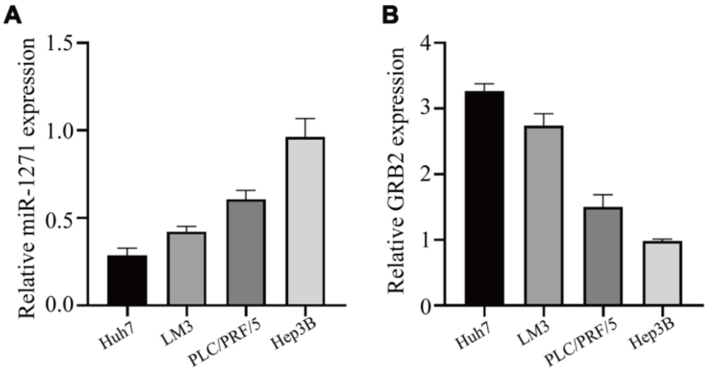

Supplementary Figure 1. MiR-1271 and GRB2 expression in HCC cells. (**A**, **B**) QRT-PCR analysis of (**A**) miR-1271 and (**B**) GRB2 levels in HCC cell lines, Huh7, LM3, PLC/PRF/5 and Hep3B.
